# Supplementary material for: Patterns of Intron Gain and Loss in Fungi
Source: PLoS Biol. 2004 Nov 30;2(12):e422. doi: 10.1371/journal.pbio.0020422 (PMC532390; doi:10.1371/journal.pbio.0020422)
Supplement: Table S1 — Also available at http://genes.mit.edu/NielsenEtAl/. (4.3 MB ZIP). [file pbio.0020422.st001.zip › NielsenEtAl/html/578.html]

AN3085.1.NCU05818.1.MG08326.1.FG09881.1


```
 CLUSTAL W (1.82) Multiple Sequence Alignments - Introns Inserted


Sequence 1: NCU05818.1	507 aa
Sequence 2: MG08326.1	471 aa
Sequence 3: FG09881.1	425 aa
Sequence 4: AN3085.1	450 aa
Alignment Length: 516 aa
Number Identitical Residues: 189 aa
Alignment Score (without introns) 9225


MG08326.1 	MSS--QPQRPAGPVRTQSASSFAGAFDVARHKLDKQ---------------LDKEMRNEL
NCU05818.1	MSCSLAPQRPGMPARQVSASSFAEEFDAARNRLTKLDFEQEQEHEPG----HDHEDESPM
FG09881.1 	MSS--------------QQTEFFKDNLPDHGEVTTP--------------------DSVP
AN3085.1  	MSVYSQQSYTDAISNGNRYRRLSNNAAERADLFTGPNVLVPPKHLMASSQPADLAVEMEM
          	**     . .           :          .   .     .   .::.. .       

MG08326.1 	KSLALVESDSSTPTSPGSLASDSGSGTPNSPGSPLTAALTPLSDPDVAEEFAFAFDIDGV
NCU05818.1	ASPSLSSSDSLTSEEPSPLESDNGE---NDDATPFTPMVDPDSD-NVADNFAFAFDIDGV
FG09881.1 	SSPELGSSSS--SDSIASLGS---------PASPLSPAVS----QPVADSFVFAFDIDGV
AN3085.1  	AKQRMESDTSSTASAESPLAASP-------ATSPLIPAST-----TTTDEFALAFDIDGV
          	 .  : .. * :.   ..* :.          :*: .         .::.*.:*******

MG08326.1 	LIRGGKVIPEAVEAMKVLNGENKYGVKV2PYIFLTNGGGKTEAERCKDLSAQLEIEVSTG
NCU05818.1	LVRGGKPIPEAIEAMKVLNGENEYGIKV2PYIFLTNGGGKFESERCADLSRQLDMTVSEG
FG09881.1 	LVRGGRAIPEALQAMKVLNGENEYGIQV2PHIFLTNGGGKTEEERCNDLSGQLQCDIKPG
AN3085.1  	LIRGGKPIPAAIEAMKYINGDNPYGVKV2PYIFVTNGGGKTEEERCLDLSRQLELEVAPG
          	*:***: ** *::*** :**:* **::* *:**:****** * *** *** **:  :  *

MG08326.1 	QFICGHTPMRELAEEHR--TVLVVGGEGEKCREVAEGYGFRDVITPGDIIKANAATAPFR
NCU05818.1	QFICGHTPMRELSSRYRDCSVLVVGGEGETCRLVAESYGFHDVITPGDILKANASTAPFR
FG09881.1 	QFICGHTPMREMAEKYG--TVLVVGGEGEKCRHVAEGYGFKDVVTPGDIIKHNAATTPFR
AN3085.1  	QFICGHTPMKEMAQKYD--TVLVVGGEGEKCRIVAEGYGFRNVITPGDFIKTHHDTTPFR
          	*********:*::..:   :*********.** ***.***::*:****::* :  *:***

MG08326.1 	KLTPQEHSNSRDLLARTGG-------------DLSKIVIEAVFVFADSRDWASDLQIIID
NCU05818.1	RLTPAEHAASRDLLFSRGRGRRHRRHRPEVVRKLSDIVISAVFVFADSRDWASDLQIILD
FG09881.1 	KLTPEEHRNSRER-------------------DFTDVVIDAVFVFADSRDWAGDIQIMLD
AN3085.1  	KLTEEEYNNSRVL-------------------DLETLRIDAIFVFADSRDWAGDQQIILD
          	:**  *:  **                     .:  : *.*:**********.* **::*

MG08326.1 	VAMSKGGRLETRSETFDEGPPVYFSHNDTLWAAQHEHARLGMGALRRIVEVVFSDVTGGK
NCU05818.1	VAQSRGGRLETRSETFDEGPPIYFSHSDVVWSAAHEHVRLGMGALRKMVETVFEETTGGK
FG09881.1 	VAMSKGGRLGTRSETNDEGPPFYFSHNDVVWSAAHEHVRLGMGALRRMFEVTFEDLTGGN
AN3085.1  	CLMSKNGNMLERSTTFDEGPPVFFSHNDVVWSTSHQHSRIGMGALRASLEALYKAVTGKE
          	   *:.*.:  ** * *****.:***.*.:*:: *:* *:******  .*. :.  ** :

MG08326.1 	GTLRAHSFGKPQVPTFEFATRLLQRWREDRHGLTSG-------APPPETVYFVGDTPESD
NCU05818.1	-KLVTHAFGKPQVGTFEFAERLLRQWRAEKHGLGLGTLHGRSQKPAVRTVYFVGDTPESD
FG09881.1 	GVLHTHAFGKPQVSTFEFASRLMGQWRQTEHGLVAP----------PDTVYFVGDTPESD
AN3085.1  	--LTTVAFGKPQLGTYEFATRLLRQWRKEFHGINKP----------PKTVYFVGDTPESD
          	  * : :*****: *:*** **: :**   **:               ************

MG08326.1 	IRGTNAMDAVSE---NEWYSILVKTGVYQDGTVPKYVPRVTVDTVLDAVNHGIEREMERR
NCU05818.1	IKGTNAMDEKSGREGTEWYSILVKTGVYQEGTEPRYRPRKLVDNVLDAVHHGIRREMARK
FG09881.1 	IRGTNAVNKVAD---NDWYSILVKTGVYQEGTEPAYKPRVTVQNVLDAVNHGIKREMDKK
AN3085.1  	IRGTNKFNEISD---TDWFSILVKTGVYQEGTIPRYPPKKTCGTVLDAVKFAINRELG--
          	*:*** .:  :    .:*:**********:** * * *:    .*****:..*.**:   

MG08326.1 	KAAALAAPAVAAEKGIK-MQLTEQENAVEHLEVVA--
NCU05818.1	RTTSGGRLLSLDEDVFKNVGERRMPEIVERVGPFSEQ
FG09881.1 	AAKG----QMIKPALIPDSGITEIAVDMDGITI----
AN3085.1  	---------------LKTDDLRNNEPSPERYGEVRA-
          	               :      .     :
```
